# Outcome of Cardiac Monitor During Sleep Study for Screening of Subclinical Atrial Fibrillation

**DOI:** 10.7759/cureus.8987

**Published:** 2020-07-03

**Authors:** Naeem Alshoaibi

**Affiliations:** 1 Medicine, King Abdulaziz University, Jeddah, SAU

**Keywords:** obstructive sleep apnea, sleep-disordered breathing, apnea–hypopnea index, atrial fibrillation, arrhythmia, cardiac rhythm monitoring

## Abstract

Background

There is growing evidence of a strong association between obstructive sleep apnea (OSA) and cardiovascular co-morbidities including atrial fibrillation (AF). We wanted to assess the usefulness of the overnight cardiac monitoring to screen for AF during the sleep study in patients newly diagnosed with OSA, in order to establish the usefulness of overnight active screening for subclinical AF during the sleep study in these patients.

Methods

A retrospective study in patients with new diagnosis of OSA carried out between January 2014 and December 2019 in the sleep clinic at King Abdulaziz University Hospital, Jeddah, Saudi Arabia. All patients newly diagnosed with OSA (apnea-hypopnea index >5) were selected to undergo a clinical questionnaire regarding symptoms, co-morbidities and risk factors. Subjects with history of cardiac arrhythmias or having anti-arrhythmic treatment were excluded. Eligible patients underwent an overnight rhythm monitoring to screen for AF or any rhythm disturbance.

Results

We included 250 respective patients with OSA, 54% were males and 82% aged more than 35 years. The majority of patients were married (83%), of Saudi nationality (81%), and 90% were overweight or obese, apnea hypopnea index (AHI) was mild (5-14) in 30%, moderate (15-29) in 38% and severe (30 or more) in 32% of the patients. No cardiac arrhythmia was detected in all the study population, while only two patients complained of palpitations and was due to sinus tachycardia. Assessment of other risk factors showed 26% cases of diabetes mellitus, 39% of hypertension, 1% of renal failure, 9% of ischemic heart disease, 17% of thyroid dysfunction, 6% of stroke and 4% of dyslipidemia.

Conclusion

The findings of this study show null incidence of cardiac arrhythmia during the apnea-hypopnea episodes in a cohort of patients with confirmed OSA. However, in view of the frequently reported association, the screening for subclinical atrial fibrillation needs long-term rhythm surveillance and should be targeted to symptomatic patients.

## Introduction

Obstructive sleep apnea (OSA) is characterized by repetitive closure of the upper airway leading oxygen desaturations and sleep disturbance causing variety of symptoms such as snoring and daytime fatigue and tiredness [1]. It is estimated to be relatively frequent disorder in Saudi Arabia, especially in patients with cardiovascular co-morbidity [2,3]. In primary care setting, one in three middle-aged Saudi males were reported to be at risk of OSA [2,4].

The epidemiological associations of OSA with several emerging demographic and metabolic factors, such as obesity, increasing age, and glucose metabolic disorders may predict a dramatic increase in the incidences of OSA in the future [1,4-7].

Owing to its close association with increased risk of sudden death and high cardiovascular morbidity including atrial fibrillation (AF), OSA is considered to be one of the most severe types of sleep-related breathing disorders [5,8,9]. Although, the association of OSA with cardiac arrhythmias was evidenced since late 1970s, the exact pathophysiological mechanisms are still uncertain [10]. However, several pathophysiological mechanisms associated with OSA are considered for either triggering or maintaining the AF. Frequent hypoxemia, oscillations in intrathoracic pressure, sympathetic and parasympathetic changes, systemic hypertension, and cardiac diastolic dysfunction, all considered as a mechanistic risk factors for atrial fibrillation [11].

Hypoxemia consequent to the characteristic episodes of airway obstruction may lead to bradycardia, peripheral vasoconstriction and decreased myocardial oxygen consumption, as a result of a cardiac parasympathetic stimulation. At relief of the obstruction, a reflex increase in sympathetic activity is produced, causing dramatic increase in cardiac rhythm, concomitant to a compensating hyperpnea. Other mechanisms including, high blood pressure and acute or chronic atrial enlargement are suggested to contribute in the incidence of AF during OSA episodes [8].

## Materials and methods

Objectives

This study aims to establish the usefulness of overnight active screening for subclinical AF during sleep study, in patients newly diagnosed with OSA and who have no history of arrhythmia.

Methods

This was a retrospective study carried out between January 2014 and December 2019 in the sleep clinic at King Abdulaziz University Hospital, Jeddah, Saudi Arabia. A cohort of study patients were randomly sampled who were newly diagnosed with OSA (apnea-hypopnea index ≥ 5). The patients underwent a clinical questionnaire regarding symptoms, co-morbidities and risk factors of AF. Table 1 presents the clinical questionnaire used in this study.

**Table 1:** Clinical questionnaire used in the study.

Name: File number: Weight: ... kg, Height: ... cm, BMI: ...	
Age:	□ <18 □ 18-<35 □ 35-<50 □>50
Gender:	□ Male □ Female
Marital status	□ Married □ Single □ Divorced □ Widowed
Nationality	□ Saudi □ Others (............ )
Palpitation	□ Yes □ No
Pulse:	□ Rate: ... Regular Rhythm: □ Yes □ No
Chronic diseases	□ DM □ HTN □ Dyslipidemia □ IHD □ Thyroid disease □ Atrial Fibrillation □ Stroke □ Renal Failure □ Other ......
Cardiac monitor	□ Yes □ No
Apnea hypopnea index (AHI)	□ Mild □ Moderate □ Severe

Those with a history of arrhythmia, as well as those having anti-arrhythmic treatment, were excluded from the study. Eligible patients underwent a cardiac rhythm monitoring to screen for AF. The study was performed according to the Helsinki declaration ethical guidelines and is approved by the institutional review board of King Abdulaziz University Hospital.

Apnea-hypopnea index and diagnosis of obstructive sleep apnea

The AHI is defined by the hourly average number of airflow interruption (apnea) and reduction (hypopnea) events occurring during sleep. A value ≥ 5 events per hour is generally diagnostic for OSA, the severity of which is proportional to the AHI value [7,12].

Cardiac rhythm monitoring

A continuous recording of the cardiac rhythm was achieved for the patients with usual activity to assess the incidences of arrhythmia episodes, either during or out of sleep [13]. The outcomes were denoted by “1” and “0”, where “1” indicating the detection of an arrhythmia, while “0” indicating its absence on the recording.

Statistical methods

Statistical analysis was performed using Statistical Package for Social Sciences version 16.0 for Windows (SPSS Inc., Chicago, IL, USA). Minimum (n = 100) target sample size was calculated for an estimated proportion of AF in OSA = 0.043, a confidence level = 0.95 and precision of estimate = 0.05 and was increased to n = 250. Descriptive statistics were used to determine the frequencies and percentages of categorical variables and means and standard deviations of continuous variables.

## Results

Demographic characteristics

The present study included 250 patients diagnosed with OSA. The participants were mostly adults, aged ≥35 years (82%). Fifty-four percent of the participants were male. The majority of patients were married (83%), of Saudi nationality (81%) and were overweight or obese (90%). Apnea hypopnea index (AHI) was severe (≥30) in 32% of the patients. Table 2 presents demographic characteristics of the study population.

**Table 2:** Demographic characteristics of the study population (N = 250). *SD = standard deviation

Parameter	Value	Frequency	%
Age	<18	4	1.6
	18 - < 35	41	16.4
	35 - < 50	93	37.0
	≥50	112	45.0
Gender	Male	135	54.0
	Marital status	Married	207	83.0
Single		35	14.0
Divorced		6	2.4
Widowed		2	0.6
Nationality	Saudi	202	81.0
	Non-Saudi	48	19.0
Height (mean {SD}, cm)	Range (164 {69-190})	160.01	18.05
Weight (mean {SD}, kg)	Range (103 {44-210})	107.62	32.81

No cases of cardiac arrhythmia or AF were detected in the study population, while only two patients complained of palpitations and it was due to sinus tachycardia. Assessment of other risk factors showed 26% cases of diabetes mellitus, 39% of hypertension, 1% of renal failure, 9% of ischemic heart disease, 17% of thyroid dysfunction, 6% of stroke and 4% of dyslipidemia. Table 3 represents the results of the risk factors assessment in the study population.

**Table 3:** Results of the risk factors assessment and cardiac rhythm recordings in the study population.

Parameter	Value	Frequency	%
Risk factors			
Palpitation	No	248	99.0
	Yes	2	1.0
Body mass index	Underweight < 18.5	5	2.0
	Normal weight 18.5 - 24.9	20	8.0
	Overweight 25 - 29.9	35	14.0
	Obesity ≥30	190	76.0
Apnea Hypopnea Index	5 - 14 mild sleep apnea	75	30.0
	15 - 29 moderate sleep apnea	95	38.0
	≥30 severe sleep apnea	80	32.0
Diabetes Mellitus		65	26.0
Hypertension		97	39.0
Renal failure		2	1.0
Ischemic Heart Disease		23	9.0
Thyroid dysfunction		42	17.0
Stroke		15	6.0
Dyslipidemia		10	4.0
Outcome			
Cardiac rhythm	Sinus rhythm	250	100.0
	Arrhythmia	0	0

## Discussion

This study showed that the overnight monitor during the sleep study is not enough to detect AF in patients with OSA. Frequent surveillance and long-term monitoring are the best way to address the diagnosis of paroxysmal AF in patients with OSA.

Prevalence of AF in general population

According to Go et al., the prevalence of diagnosed AF (USA) was 0.95% (CI = 95%, 0.94% - 0.96%) and men were more prone to AF than women (1.1% vs 0.8%; P < 0.001). Aging is one of the most common risk factors of AF, the prevalence around 9% in people of age ≥ 80 years [14]. Davis et al. conducted a study in 3960 participants in general population to evaluate the prevalence of AF. AF was found in 78 of the random population (2.0%; 95% CI 1.6% - 2.4%). Prevalence was 1.6% in women and 2.4% in men and was found to increase with age. The increase in prevalence was 0.2% to 8.0% from the age group of 45-54 years and >75 years, respectively [15]. Norberg et al., in a study conducted in general population of Sweden revealed that the prevalence of AF was 3.0% (3.4% in men and 2.6% in women). AF prevalence increased steadily with age, and was 16.8% in patients aged ≥75 years and 21.9% in people ≥85 years old [16].

Prevalence of subclinical AF in general population

In the STROKESTOP (Sweden) observational study of 7173 individuals 75 to 76 years of age in Sweden, previously unknown AF was detected using intermittent electrocardiographic recordings over three weeks in 3% [17]. The Atrial High Rate Episodes (A-HIRATE) study showed that the incidence of AHRE was 89% and 49% in patients with previous atrial tachyarrhythmias and in patients with no history of atrial tachyarrhythmias, respectively. Quirino et al. showed that the incidence of AF was 74%. Both these studies showed that most of the episodes were asymptomatic [18].

A study showed that 700,000 people in the United States may have undiagnosed AF, with an estimated cost burden of 3.2 billion dollars [19,20]. It is well known that asymptomatic AF is associated with similar risk of all-cause death, cardiovascular death, and thromboembolism compared to symptomatic AF [21].

Prevalence of AF in patients with OSA

The prevalence of OSA is significantly higher in patients with AF when compared to the general population. Gami et al. identified that 17% of AF patients have OSA [11]. Similarly, Bitter et al. established that the prevalence of OSA was 42.7% among consecutive patients with AF [22]. This prevalence was even higher in a study by Braga et al., who found OSA in 81.6% of patients with AF [23]. Similarly, OSA (defined by an apnea-hypopnea index ≥ 5) was demonstrated to be a strong predictor of incident AF increasing the odds of AF by a factor of 2.18, as AF occurred in 4.3% of patients with OSA and in 2.1% of those without OSA [11]. Furthermore, in the Sleep Heart Health Study, an increase of four-fold risk for AF was observed in patients with severe OSA (apnea/hypopnea index [AHI] > 30 events/hr) [24]. OSA is affecting 10-15% of the general population and almost share the same risk factors of the AF [25]. Obesity is a global public health problem and it is the most common metabolic disease that can lead to AF and OSA, almost 35% of the adult Americans suffer from obesity. Hence AF and OSA will impact the global health care [26].

Diastolic dysfunction increases the risk of AF by increasing the left atrial size, stretching the atrial wall, changes the cardiac transmural pressure and increases the cardiac wall stress [27]. Studies have shown the independent association of OSA and diastolic dysfunction [28,29].

## Conclusions

The findings of this study reject the usefulness of the short-term overnight cardiac monitoring in a cohort of asymptomatic patients newly diagnosed with OSA. However, in view of the frequently reported association, evocative symptoms of AF should be actively searched through comprehensive history, and suspected patients should benefit from long-term rhythm surveillance to address the diagnosis. Furthermore, several risk factors have been identified in the selected population and follow-up of these subjects might provide evidence of AF symptoms in the long-run. There are very limited studies on subclinical AF in patients with OSA and more studies of this nature are required for a proper conclusion in this aspect.
